# Cancer stem cells are enriched in Fanconi anemia head and neck squamous cell carcinomas

**DOI:** 10.3892/ijo.2014.2677

**Published:** 2014-09-26

**Authors:** JEAN WU, QINGSHAN MU, VARATHARASA THIVIYANATHAN, ANANTH ANNAPRAGADA, NADARAJAH VIGNESWARAN

**Affiliations:** 1 Department of Diagnostic and Biomedical Sciences, The University of Texas School of Dentistry at Houston, Houston, TX 77054, USA; 2 Department of Nanomedicine and Biomedical Engineering, The University of Texas Medical School at Houston, Houston, TX 77030, USA; 3 The Singleton Department of Pediatric Radiology, Texas Children’s Hospital, Baylor College of Medicine, Houston, TX 77030, USA

**Keywords:** Fanconi anemia, head and neck squamous cell carcinoma, cancer stem cells, Aldefluor assay, tumorspheres, xenograft

## Abstract

Fanconi anemia (FA) patients have an increased risk of head and neck squamous cell carcinoma (HNSCC) at a higher rate with no apparent risk factors. HNSCC of FA patients is an aggressive tumor characterized by multifocal origin, early metastases and frequent recurrences. Given that cancer stem cells (CSC) drive tumorigenesis, tumor recurrence and metastasis, in this study, we characterized the CSC population in FA and sporadic HNSCC. The Aldefluor assay was used to characterize and isolate CSC with high aldehyde dehydrogenase (ALDH) activity (ALDH^pos^) in cell lines derived from FA and sporadic HNSCC. Isolated ALDH^pos^ and ALDH^neg^ cells were examined for the expression of stemness genes using reverse transcription-polymerase chain reaction (RT-PCR) array. Tumor cell-derived FA and sporadic HNSCC were examined for their ability to form tumorspheres *in vitro*. Stem-like cell population in FA and sporadic HNSCC in human and mouse xenograft tumors were evaluated using ALDH isoform 1 (ALDH1) immunohistochemistry. FA-HNSCC cell lines harbor a greater proportion of ALDH^pos^ cells (15–31%) compared to sporadic HNSCC (10%). Expression of Nanog, Oct-3/4 and Stella, molecular markers of undifferentiated embryonic stem (ES) cells were detected in the ALDH^pos^ FA-HNSCC cells and not in the ALDH^neg^ cells. FA-HNSCC cell lines revealed enhanced *in vitro* tumorsphere formation compared to sporadic HNSCC cells. A higher percentage of ALDH1^pos^ tumor cells are noted in the human and mouse xenograft tumors of FA-HNSCC compared to sporadic HNSCC tumors. FA-HNSCC are highly enriched for CSC and may serve as a model to develop CSC-targeted therapies for HNSCC.

## Introduction

Fanconi anemia (FA) is a chromosomal instability disorder inherited as an autosomal- or X-chromosomal recessive trait due to germline mutations in one of 15 FA genes (FANCA/B/C/D1/D2/E/F/G/I/J/L/M/N/O/P) involved in the DNA repair pathway (1). Clinically, FA is characterized by various congenital malformations, increased risk of malignancies and progressive bone marrow failure (2). Head and neck squamous cell carcinoma (HNSCC) is the most frequently diagnosed solid cancer in FA patients. Some FA patients may not exhibit bone marrow failure due to hypomorphic mutations, or reversions or mosaicism in the hematopoietic tissue and the diagnosis of HNSCC usually precedes the diagnosis of FA in these patients (3). Oral cavity is the predominant site of HNSCC in FA patients, frequently occurring in the tongue and gingiva (3). The risk of HNSCC among FA patients is 800-fold higher than in the general population (4,5). HNSCC in FA patients occurs at a younger age (median age: 27 years) than the general population and FA patients as young as 10 years have developed HNSCC.

Hematopoietic stem cell transplantation (HSCT), which greatly extends life-expectancy, is the treatment of choice for FA patients. FA patients who survive into adulthood after HSCT are at a higher risk for developing HNSCC, significantly reducing their quality of life and overall life-expectancy (3–5). FA patients (~50%) who have not undergone HSCT are expected to develop HNSCC by 45 years of age, whereas the cumulative incidence of HNSCC in bone marrow-transplanted FA patients is estimated to be 100% at the same age (6). Increased risk for HNSCC in FA patients after HSCT is attributed to the conditioning regimen and the occurrence of chronic graft-vs.-host disease in the oral cavity (3). HNSCC in FA patients occurs without exposure to any of the known risk factors namely tobacco and alcohol (3,7). Although an association between oncogenic human papillomavirus (HPV) infection and FA-HNSCC was suggested in previous studies (8), recent studies suggest that HPV infection is not the cause for HNSCC in FA patients (9). Moreover, molecular profiles of FA-HNSCC cells are not significantly different from those of sporadic HNSCC (10). These findings indicate increased susceptibility to a variety of mutagens and resultant non-lethal DNA damage that promotes oral mucosal tumorigenesis in FA patients (11).

Gammon *et al* reported the presence of a stem-like cell population in FA oral cancer cell lines based on the difference in the colony morphologies between sporadic and FA-HNSCC cell lines (12). Stem-like cells, known as cancer stem cells (CSC), that initiate and sustain tumor growth and spread have been identified in a number of solid malignancies (13). A subpopulation of cells within a tumor that has a higher-tumor repopulating potential is identified as CSC (14,15). CSC have the capacity to self-renew and to give rise to heterogeneous lineages of cancer cells that populate the tumors (15). CSC share gene expression profiles and phenotypic characteristics with embryonic and somatic stem cells including a slow proliferation rate and resistance to standard chemotherapy and radiation therapy (16). Tumors with a higher fraction of CSC exhibit therapeutic resistance and increased risk for local recurrence and distant spread (16,17). CSC can be identified and isolated using various markers and CD44 expressing tumor cells isolated from HNSCC were identified as CSC based on increased clonogenic potential and tumor-forming ability (18,19). Recently, HNSCC cells expressing high levels of aldehyde dehydrogenase (ALDH) were identified as CSC (20). The Aldefluor assay is considered a reliable method to enrich and propagate CSC in various solid cancers including HNSCC (20). The Aldefluor assay measures ALDH activity by quantifying the conversion of ALDH substrate, BODIPY aminoacetaldehyde to a fluorescent reaction product BODIPY aminoacetate (21). Aldefluor-treated tumor cells with high ALDH isoform 1 (ALDH1) activity turn brightly fluorescent and two subpopulations (ALDH^pos^ and ALDH^neg^ cells) can be enumerated by standard flow cytometer or isolated by fluorescence-assisted cell sorting (FACS) for further analysis. Similarly, immunohistochemical staining using an ALDH1-specific antibody has been used successfully to identify and quantify CSC in formalin-fixed paraffin-embedded tumor sections. Aldefluor assay and ALDH1 immunohistochemistry are widely used for detection and enumeration of CSC in tumor cell lines and tumor samples, respectively (22–24). In this study, we used the Aldefluor assay, ALDH1 immunohistochemistry and tumorsphere-formation to quantify and characterize CSC populations in FA and sporadic HNSCC cell lines and tumor samples. We analyzed the expression patterns of 14 stemness-related genes in ALDH1^pos^ and ALDH1^neg^ cells isolated from FA-HNSCC cells using reverse transcription-polymerase chain reaction (RT-PCR).

## Materials and methods

### Cell culture

The human FA-HNSCC cell lines VU-1365 and VU-1131 were kindly donated by Dr Ruud H. Brakenhoff (Vrije University Medical Center, Amsterdam, The Netherlands) and OHSU-974 cell line was obtained from Dr Laura Hayes (Oregon Health and Science University, Portland, OR, USA). UMSCC-22A, a human sporadic HNSCC cell line, was obtained from Dr Thomas E. Carey, University of Michigan. Molecular phenotypes of these cell lines have been defined in published reports and are shown in Table I (10,25). These cell lines were grown in adherent conditions using the recommended culture medium (10,25).

### Human and xenograft FA-HNSCC tumor samples

Formalin-fixed paraffin-embedded tissue sections of FA-HNSCC specimen and its corresponding orthotopic tongue xenografts were kindly gifted by Dr Susanne Wells (Cincinnati Children’s Hospital, Cincinnati, OH, USA). FA-HNSCC tumor sample of a FANC-B deficient 56-year old female was obtained through the National Disease Research Interchange (NDRI no. 0066421; PD-RD-000237). Tumor cells derived from the fresh tumor of the same patient (FAHNSCC-2) were implanted into the tongue of NOD/SCID mice to generate the orthotopic tumor xenografts.

### Aldefluor assay and FACS of ALDH^pos^ and ALDH^neg^ cells

Tumor cell fractions with high (ALDH^pos^) and low (ALDH^neg^) ALDH activity among FA (VU-1131, VU-1365 and OHSU-974) and sporadic (UMSCC-22A) HNSCC cell lines were quantified using the Aldefluor kit (StemCell Technologies, Vancouver, BC, Canada) according to the manufacturer’s protocol. Briefly, FA and sporadic HNSCC cells (1×10^6^ cells/ml) were resuspended in Aldefluor assay buffer containing ALDH1 substrate BAAA without (test sample) and with ALDH1 inhibitor DEAB (negative control). Test sample and negative control were incubated for 45 min at 37°C and then the cells were centrifuged and resuspended in an Aldefluor assay buffer and kept on ice for FACS. The amount of fluorescent ALDH1 reaction product produced in the cells is proportional to their ALDH1 activity and only viable cells with intact cell membrane will retain the fluorescent reaction product. The intensely fluorescent (ALDH^pos^) cells were detected in the green fluorescent channel (FITC; 520–540 nm) and calculated as the percent of ALDH^pos^ in each cell line. The sorting gates were generated using the DEAB-treated Aldefluor-stained negative control cells.

The FA-HNSCC cell line VU-1365 with the highest ALDH^pos^ fraction was used for isolating ALDH^pos^ and ALDH^neg^ cells by FACS using a FACSAria II (BD Biosciences, San Jose, CA, USA). Isolated Aldefluor positive and negative fractions were used for RNA extraction and RT-PCR analysis of a set of genes linked to ‘stemness’ phenotype.

### RT-PCR analysis of stemness gene expression in ALDH^pos^ and ALDH^neg^ cells

Expression patterns of 14 genes that are commonly used as molecular markers for undifferentiated (DPPA5/ESG1, Nanog, Oct-3/4, SOX2) and lineage-committed (Nestin, Otx2, TP63, AFB, GATA-4, PDX-1, SOX17, HNF-3β, Brachyury, Stella) embryonic stem cells (ESC) and induced pluripotent stem (iPS) cells were examined using the Human Pluripotent Stem Cell Assessment Primer Pair Panel kit (R&D Systems, Minneapolis, MN, USA) according to the manufacturer’s protocol. Briefly, total RNA was extracted from ALDH^pos^ and ALDH^neg^ cells isolated from FA-HNSCC cell line VU-1365 using RNeasy^®^ Mini kit (Qiagen, Valencia, CA, USA). RNA was treated with DNase I (Ambion, Austin, TX, USA) to remove DNA contamination and absence of genomic DNA was further confirmed by running a control reaction of PCR of total RNA without reverse transcription (RT). RNA concentration was measured using a NanoDrop ND-1000 spectrophotometer (NanoDrop Technologies, Houston, TX, USA) and 1 μg RNA of each sample was used for cDNA synthesis using the Cells-to-cDNA™ II kit (Ambion/Applied Biosystems, Austin, TX, USA). cDNA (1 μl) was used for each primer pair, and PCR amplification was performed according to the manufacturer’s recommended parameters. Amplified PCR products were analyzed on a 1.8% agarose gel and the predicted sizes of the PCR products range from 230–591 bp. Synthetic double-stranded DNA provided in the kit (positive control 57) was used as a positive control for PCR amplification for each pair of primers. Human GAPDH primer is used as a control for successful cDNA synthesis.

### Tumorsphere formation assay

Tumorsphere-forming potential of UMSCC-22A and VU-1365 cells were assayed using the MammoCult™ kit (Catalog no. 05620; StemCell Technologies) according to the manufacturer’s protocol. Briefly, 80–90% confluent tumor cells were harvested using a sterile cell scraper without trypsinization and resuspended in complete MammoCult^®^ medium and centrifuged the cells at 500 × g for 3 min at room temperature. Cell pellets were resuspended in complete MammoCult^®^, counted and diluted in the same media to a predetermined concentration. Cells were plated in triplicates (5,000 cells/well) in a 6-well ultra-low adherent plate (Catalog no. 27145; StemCell Technologies). Cells were grown at 37°C and 5% CO_2_ for 7 days in a tissue culture incubator and monitored daily for tumorsphere formation. After 7 days, photomicrographs of tumorspheres were taken using an inverted phase-contrast microscope and used for counting the number of tumorspheres per high power field (HPF).

### ALDH1 immunohistochemistry

Archival specimens of formalin-fixed paraffin-embedded sporadic HNSCC (n=5), FA-HNSCC (n=1) and orthotopic FA-HNSCC xenografts (n=2) generated in the tongue of NOD/SCID mice were used to evaluate the expression of ALDH1 in tumor cells. Representative tissue sections were deparaffinized and rehydrated and antigen retrieval was performed by boiling in Antigen Decloaker (Biocare Medical, Concord, CA, USA) solution for 2 min. Endogenous peroxidase activity was blocked by incubating the tissue section with 3% H_2_O_2_ in methanol for 10 min. Non-specific binding sites were blocked by incubating the tissue sections in Background Terminator (Biocare Medical) solution for 10 min. Tissue sections were treated with normal horse serum for 10 min to block non-specific binding sites and then incubated with anti-human ALDH-1 antibody (1:100; BD Transduction Laboratories, mAb Clone no. 44) overnight at room temperature. ALDH1-antibody binding sites were detected using the Vectastain Elite ABC kit-Mouse (Vector Laboratories, Inc., Burlingame, CA, USA) according to the protocol provided with this kit. Peroxidase reactivity was visualized using 3-3′-diaminobenzidine tetrachloride as chromogenic substrate and counterstained with hematoxylin. For negative control, tissue sections were incubated with mouse IgG1 isotype control.

## Results

### ALDH1-^hi^ subfraction is enriched in FA-HNSCC cell lines

**Figure 1:**
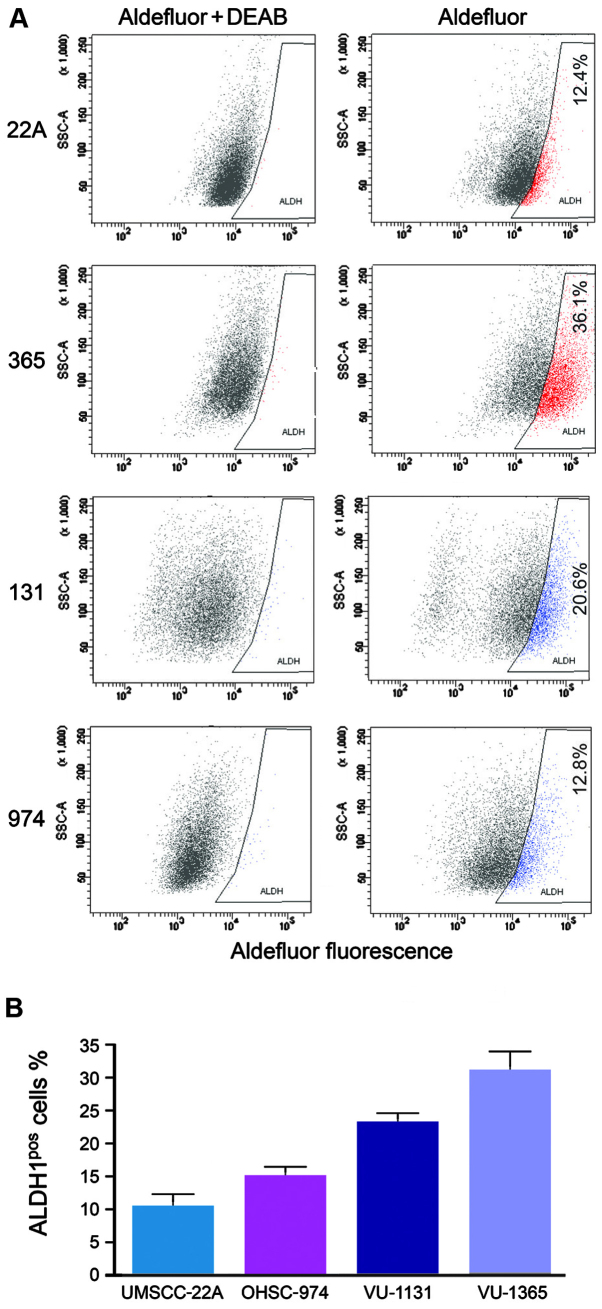
Aldefluor positive and negative cell populations in sporadic (UMSCC-22A) and FA-HNSCC cell lines. (A) Representative fluorescence-assisted cell sorting (FACS) plots of head and neck squamous cell carcinoma (HNSCC) cell lines stained with Aldefluor reagent with and without the DEAB inhibitor and (B) the bar graph reveals greater proportion of (13–31%) ALDH isoform 1 (ALDH1) positive cells in FA-HNSCC compared to sporadic HNSCC (12%).

Using the Aldef luor assay, we examined the ALDH^pos^ and ALDH^neg^ fractions in sporadic (UMSCC-22A) and FA (VU-1365, VU-1131 and OHSU-974) cell lines. FACS analysis demonstrated variable ALDH1 expression among sporadic and FA-HNSCC cell lines. FA-HNSCC cell lines consistently demonstrated a greater fraction of ALDH^pos^ cells (VU-1365=31±2.9%, VU-1131=23±2.7%, OHSU-974=15±2.0%) compared to sporadic HNSCC cell line (UMSCC-22A=10.33±3.0%) (Fig. 1). The FA-HNSCC cell line VU-1365 demonstrated the highest percentage of ALDH^pos^ CSC. We selected this cell line to isolate the ALDH^pos^ and ALDH^neg^ fraction to confirm the stemness phenotype of ALDH^pos^ cells by RT-PCR.

### ALDH1^pos^ and ALDH1^neg^ cells isolated from FA-HNSCC differentially express stemness-related genes

**Figure 2:**
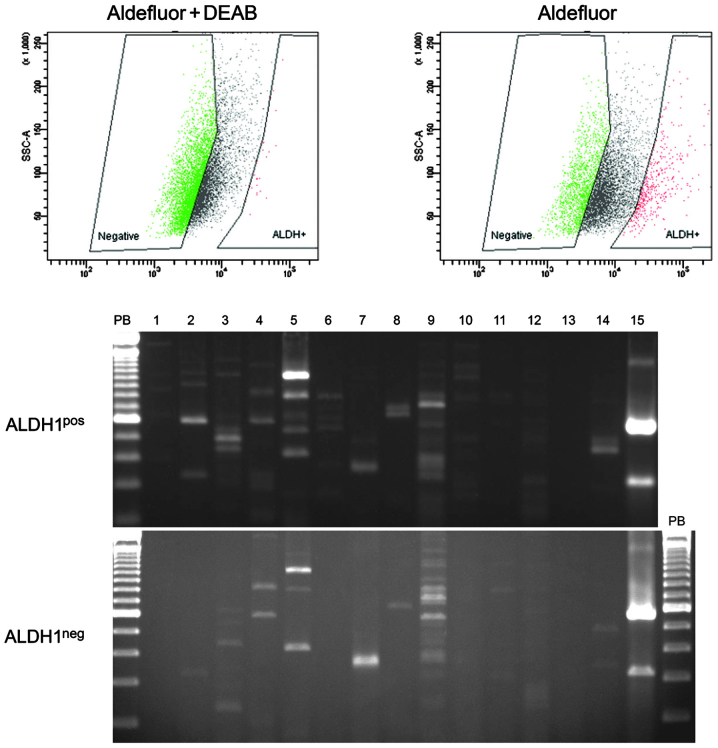
The expression profiles of stemness-genes in ALDH1^pos^ and ALDH1^neg^ cell populations in FA-HNSCC. The Aldefluor assay was used to isolate ALDH1^pos^ and ALDH1^neg^ cells in VU1365 cell line and used for RNA isolation and reverse transcription-polymerase chain reaction (RT-PCR) array analysis. The human pluripotent stem cell assessment primer pairs were used to amplify the mRNA transcripts of 14 genes that are frequently used as molecular markers of undifferentiated embryonic stem (ES) cells (Lane 2, DPPA5/ESG1; Lane 3, Nanog; Lane 4, Oct-3/4; Lane 5, SOX2), ectodermal lineage-committed stem cells (Lane 5, SOX2; Lane 6, Nestin; Lane 7, Otx2; Lane 8, TP63), endodermal lineage-committed stem cells (Lane 9, AFB; Lane 10, GATA-4; Lane 11, SOX17; Lane 12, HNF-3β), mesodermal lineage-committed germ cells (Lane 13, Brachury) and germs cells (Lane 14, Stella). Lane 15, GAPDH-loading control.

To determine whether ALDH1^pos^ isolated from FA-HNSCC cells represent tumor cells with stem cell-like properties, we compared the expression profiles of genes linked to undifferentiated and lineage-committed ECS and iPS cells between ALDH1^pos^ an ALDH1^neg^ cells using RT-PCR. The ALDH1^pos^ cells consistently express mRNA transcripts of genes specific for undifferentiated ESC and iPS cells (Nanog, Oct-3/4) and germ cells (Stella), whereas these genes are not expressed in ALDH1^neg^ (Fig. 2). Both ALDH1^pos^ and ALDH1^neg^ express SOX2, Nestin and TP63 genes that are linked to ectodermal lineage-committed ECS and iPS cells. Neither of these fractions express genes linked to either endodermal-(AFB, GATA-4, PDX-1, SOX17 and HNF-3β) or mesodermal-(Brachyury) lineage-committed ES and iPS cells (Fig. 2).

### FA-HNSCC cells reveal higher tumorsphere formation capacity than the sporadic HNSCC cells

**Figure 3:**
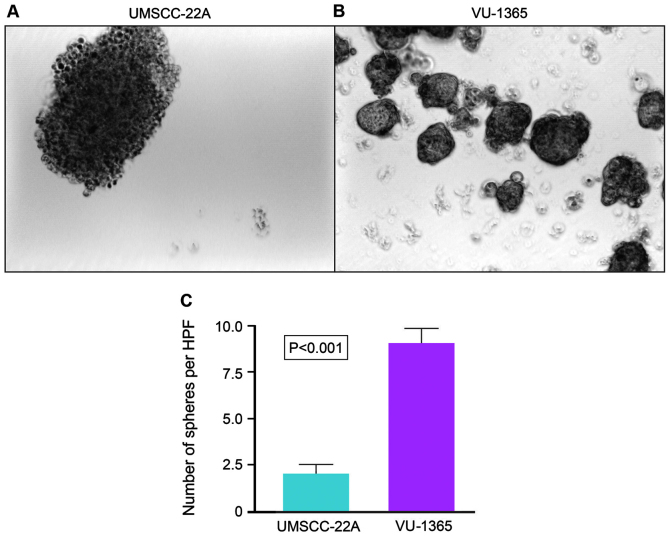
Representative images of the tumorsphere size, number and distribution formed by (A) sporadic (UMSCC-22A) and (B) Fanconi anemia (FA) (VU-1365) head and neck squamous cell carcinoma (HNSCC) cell lines. (C) Quantification of number of tumorspheres formed by FA-HNSCC cells were significantly higher (p<0.001) than those of sporadic HNSCC cells.

Sphere-forming capacity of tumor cells correlates directly with CSC phenotype and thus tumorigenic efficiency of the cancer cells can be determined based on the number of spheres that originate from specific number of seeded cells. We compared the tumorsphere-forming capacities of tumor cell lines derived from FA-HNSCC (VU-1365) and sporadic HNSCC (UMSCC-22A). Although both cell lines formed stable tumorspheres at day 7, there were significant quantitative and qualitative differences in their sphere-forming potential (Fig. 3). The number of tumorspheres formed by VU-1365 cells are significantly (p<0.001) higher than UMSCC-22A cells (Fig. 3). Tumorspheres formed by VU-1365 cells appear to be solid and round whereas UMSCC-22A spheres are looser, less cohesive and irregular in shape (Fig. 3).

### Expression of ALDH1 in FA and sporadic HNSCC tumor samples

**Figure 4:**
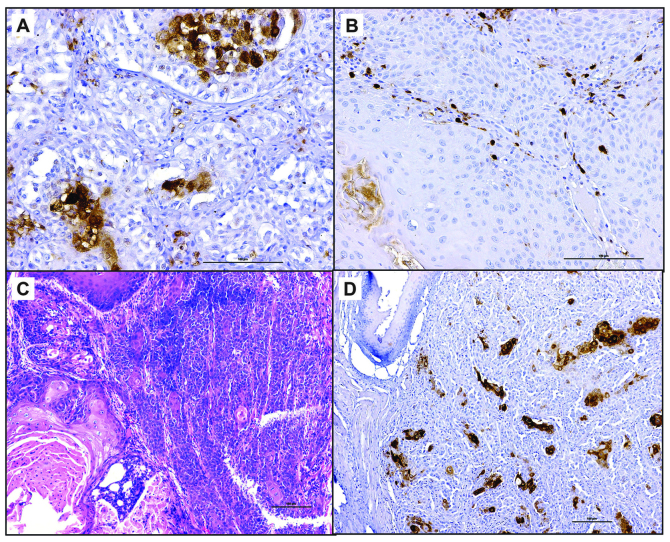
Representative images of ALDH1^pos^ cells in (A and D) Fanconi anemia (FA), and (B) sporadic, (A and B) human primary, and (C and D) mouse xenograft head and neck squamous cell carcinoma (HNSCC) tumor sections. Both (A) human primary and (D) mouse xenograft FA-HNSCC tumor sections exhibit greater fraction of ALDH1^pos^ cells than (B) sporadic HNSCC tumor section. Immunohistochemical staining for ALDH isoform 1 (ALDH1) in (A and B) primary and (D) xenograft tumor sections. (C) Hematoxylin and eosin stained section of orthoptic mouse tongue xenograft of FA-HNSCC. Scale bar, 100 μm.

Tumor cells positive for ALDH1 were noted in three of the five sporadic HNSCC tissue sections. Less than 10% of the tumor cells stained positive for ALDH1 in sporadic HNSCC whereas >25% of tumor cells are positive for ALDH1 in FA-HNSCC tissue section (Fig. 4). Interestingly, the FA-HNSCC tumor xenograft sections exhibited similar patterns of ALDH1 expression as its parental tumor. Moreover, ALDH1-positivity was noted in individual and small groups (3–5 cells/group) of tumor cells in sporadic HNSCC tumor sections. In contrast, multiple large foci of tumor cells express ALDH1 in FA-HNSCC sample and its corresponding tumor xenograft (Fig. 4).

## Discussion

The enzymatic activity of ALDH1, which can be measured by Aldefluor assay, has been identified as a reliable marker to identify normal and cancer stem cells (21). ALDH1 is widely used as a universal functional marker to identify and isolate tumor cells, with ‘stemness’ phenotypes in several solid malignant tumors including head and neck cancer (20). In this study, we used Aldefluor assay to determine the presence and size of the CSC fraction in a panel of cell lines derived from FA and sporadic HNSCC. Our findings provide convincing evidence that FA-HNSCC is highly enriched for CSC and confirm a previous study reporting the presence of a subpopulation of tumor cells in FA-HNSCC with stem cell-like properties (12). Evidence that ALDH1^pos^ cells in FA-HNSCC display a stem cell phenotype is further supported by their expression of genes linked to stem cell pluripotency and differentiation. We examined the expression of mRNA transcripts of 14 genes linked to undifferentiated and lineage-committed human ESC cells and iPS cells in ALDH^pos^ and ALDH^neg^ cells using gel-electrophoresis based RT-PCR. This qualitative method is convenient and easily interpretable for the presence or absence of the transcripts and a reliable alternative to microarray and quantitative RT-PCR-based gene expression-profiling assays that reveal the quantitative changes in genes that are differentially expressed. Among the 14 genes examined, transcripts for Nanog, Oct-3/4 and Stella are expressed only by ALDH1^pos^ FA-HNSCC cells and not by ALDH1^neg^ cells. The transcription factors Oct-3/4 and Nanog are crucial for the maintenance of pluripotency and self-renewal of undifferentiated ESC (26,27). Stella is a marker gene of primordial germ cells and undifferentiated ESC but not expressed by lineage-committed ESC (27). Interestingly, SOX-2 is expressed by both ALDH1^pos^ and ALDH1^neg^ cells albeit higher levels in ALDH1^pos^ cells which is in accordance with its function as a transcription factor involved in both conferring pluripotency to undifferentiated ESC and ectodermal lineage-committed ESC (27). On the other hand, TP63, a gene linked to ectodermal lineage-committed ESC, is also expressed in both ALDH1^pos^ and ALDH1^neg^ cells but its expression is more in ALDH1^neg^ cells than ALDH1^pos^ cells. Genes linked to ectodermal (Nestin, Otx2), endodermal (AFB, GATA-4, PDX-1, SOX17 and HNF-3β) and mesodermal (Brachyury) lineage-committed ESC are not expressed either by ALDH1^pos^ or ALDH1^neg^ cell fractions. Our data suggest that CSC of FA-HNSCC express the phenotype of undifferentiated ESC but not that of ectodermal lineage-committed ESC.

CSC are clonogenic and undergo asymmetric cell division resulting in self-renewal and multilineage differentiation of their progenitors (14,28). Unlimited self-renewal potential and formation of tumorspheres *in vitro* under low-attachment condition are the hallmarks of CSC and defined their tumor-initiating potential *in vivo* (29). On the contrary, cancer cells lacking the stem cell phenotype are incapable of forming tumorspheres when grown under similar culture conditions. The ability of the tumor cells to form spheres in low-attachment and serum-free culture conditions correlates with their ability to form tumors in xenograft models (29). Hence, tumorsphere-forming efficiency of cell lines correlates positively with their respective fractions of CSC and *in vivo* tumorigenicity (30). We therefore compared the tumorsphere-forming capacity of FA (VU-1365) and sporadic (UMSCC-22A) HNSCC cell lines. Our data clearly show that VU-1365 poses a higher capacity of tumorsphere formation than UMSCC-22A cells and confirms that FA-HNSCC cells contain a larger fraction of tumor cells with CSC-phenotype compared to sporadic HNSCC cells.

Next, we examined the ALDH1 expression in sporadic and FA-HNSCC tumor tissue using immunohistochemistry. ALDH1 expression was noted in all HNSCC tumor sections examined. In sporadic HNSCC tumors ALDH1^pos^ cells were scattered as single cells or small group of cells and represented a relatively small population. By contrast, FA-HNSCC tumor tissue contained a large fraction of ALDH1^pos^ cells that were distributed as sheets or islands of ALDH1^pos^ cells among the tumor tissue. Interestingly, CSC fraction and distribution pattern are very similar in FA-HNSCC tumor and in the tumor xenograft derived from the same tumor. Breast, lung, prostate and pancreatic carcinomas with a high percentage of ALDH1^pos^ cells demonstrate an aggressive clinical course with poor prognosis compared to the tumors with low ALDH1^pos^ tumor cell fraction (31–33). The relative abundance of ALDH1^pos^ CSC fraction may account for the biological differences between the FA-HNSCC and sporadic HNSCC.

HNSCC represents the most frequently diagnosed solid tumor in FA patients, developing at a significantly younger age than the general population (3,5,6). HNSCC in FA patients carries a poor prognosis and requires early intervention and aggressive surgical treatment (3). Radiation and chemotherapy have limited value in treating HNSCC in FA patients because their normal cells are highly sensitive to DNA cross-linking agents (3). Moreover, FA-HNSCC patients have a higher lifetime risk for developing multiple primary tumors than the general population due to the presence of a widespread of pre-malignant disease (3). This phenomenon commonly known as ‘field cancerization’, is used to describe multifocal pre-malignant disease, a higher than expected prevalence of second primary tumors and the occurrence of synchronous distant tumors within the upper aerodigestive tract (34). Recent studies suggest that genetic alterations that promote the development of CSC are responsible for field cancerization (35).

CSC population is either derived directly from the transformation of normal stem cells or from aberrant de-differentiation of non-CSC acquiring the molecular armaments of stem cells (14,28). In either case, mutations in the fundamental self-renewal signaling cascades lead to the formation of CSC, that are independent of regulatory pathways that govern normal stem cells (36,37). We postulate that the defect in the DNA repair pathways, a hallmark of FA, increases the risk of mutation in the genes that guard self-renewal pathways of stem cells and causes the enrichment of tumor cells with stemness in FA-HNSCC. Recent evidence points to CSC as the key drivers of field cancerization, resistance to chemotherapy and radiation therapy, disease relapse and metastasis in solid malignancies (16). It is unlikely to be a coincidence that CSC are found in abundance in FA-HNSCC with a high-risk for field cancerization and aggressive biologic behavior compared to sporadic HNSCC (3–5,38). CSC survives the conventional chemoradiation therapy and maintains their ability to re-populate to produce tumor recurrence and metastasis (39,40). Hence, CSC-targeted therapy remains an attractive approach for HNSCC in FA patients who currently have very limited treatment options.

## Figures and Tables

**Table I:** Clinical and molecular characteristics of FA and sporadic HNSCC cell lines.

Cell line	Source	Characteristics
UMSCC-22A	58YOF	Sporadic OSCC
	Oropharynx	TP53 mutation: Yes
	T2N1M0	
OHSU-974	29YOM	FA OSCC
	Tongue	FA Type: FA-A
	TNM: unknown (8)	TP53 mutation: Yes
VU-1131	34YOF	FA OSCC
	Floor of the mouth	FA Type: FA-C
	T4N2M0 (8)	TP53 mutation: Yes
VU-1365	22YOM	FA OSCC
	Oral cavity	FA Type: FA-A
	TNM: unknown (8)	TP53 mutation: Yes

FA, Fanconi anemia; HNSCC, head and neck squamous cell carcinoma.

## Abbreviations

FA: Fanconi anemia
HNSCC: head and neck squamous cell carcinoma
ALDH: aldehyde dehydrogenase
CSC: cancer stem cells
RT-PCR: reverse transcription-polymerase chain reaction
FACS: fluorescence-assisted cell sorting
